# The 3’tsRNAs are aminoacylated: Implications for their biogenesis

**DOI:** 10.1371/journal.pgen.1009675

**Published:** 2021-07-29

**Authors:** Ziwei Liu, Hak Kyun Kim, Jianpeng Xu, Yuqing Jing, Mark A. Kay

**Affiliations:** 1 Department of Pediatrics, Stanford University, Stanford, California, United States of America; 2 Department of Genetics, Stanford University, Stanford, Califormia, United States of America; University of California Riverside, UNITED STATES

## Abstract

Emerging evidence indicates that tRNA-derived small RNAs (tsRNAs) are involved in fine-tuning gene expression and become dysregulated in various cancers. We recently showed that the 22nt LeuCAG3´tsRNA from the 3´ end of tRNA^Leu^ is required for efficient translation of a ribosomal protein mRNA and ribosome biogenesis. Inactivation of this 3´tsRNA induced apoptosis in rapidly dividing cells and suppressed the growth of a patient-derived orthotopic hepatocellular carcinoma in mice. The mechanism involved in the generation of the 3´tsRNAs remains elusive and it is unclear if the 3´-ends of 3´tsRNAs are aminoacylated. Here we report an enzymatic method utilizing exonuclease T to determine the 3´charging status of tRNAs and tsRNAs. Our results showed that the LeuCAG3´tsRNA, and two other 3´tsRNAs are fully aminoacylated. When the leucyl-tRNA synthetase (LARS1) was inhibited, there was no change in the total tRNA^Leu^ concentration but a reduction in both the charged tRNA^Leu^ and LeuCAG3´tsRNA, suggesting the 3´tsRNAs are fully charged and originated solely from the charged mature tRNA. Altering LARS1 expression or the expression of various tRNA^Leu^ mutants were also shown to affect the generation of the LeuCAG3´tsRNA further suggesting they are created in a highly regulated process. The fact that the 3´tsRNAs are aminoacylated and their production is regulated provides additional insights into their importance in post-transcriptional gene regulation that includes coordinating the production of the protein synthetic machinery.

## Author summary

tRNAs were previously considered only as structural RNAs that read the genetic code from mRNAs to add the appropriate amino acids to the growing polypeptide chain. In recent years, it has been discovered that tRNAs can have non-canonical gene regulatory functions through the production of various types of tRNA-generated small RNAs (tsRNAs). We have studied tsRNAs derived from the 3´ end of the mature tRNA (3´tsRNAs). The LeuCAG3´tsRNA promotes the translation of a specific ribosomal protein mRNA by altering mRNA secondary structure, ultimately controlling ribosome biogenesis. This suggests that 3´tsRNAs may represent another means of regulating mRNA translation and protein synthesis. Additionally, the LeuCAG3´tsRNA was shown to be a useful target for treating a humanized hepatocellular carcinoma model in mice. In the current study, we establish a method showing that the 3´tsRNAs are generated from tRNAs that are aminoacylated (attached with an amino acid). Moreover, we found the concentrations of 3´tsRNAs generated in a cell are dictated at least in part by the enzymes that conjugate the specific amino acids with cognate tRNAs. This suggests 3´tsRNA generation is a highly regulated process and further points to the complexity of this additional layer of post-transcriptional gene regulation.

## Introduction

Transfer RNAs (tRNAs) are cloverleaf molecules that bring amino acids to ribosomes for translating messenger RNA into protein. In addition to their roles in protein synthesis, recent years have seen emerging evidence of noncanonical regulatory functions of tRNA and tRNA fragments in regulatory events involved with maintaining homeostasis in varying cellular environments and various diseases including cancer, neurodegenerative disease, and viral infection [1–8]. The tRNA fragments reported thus far can be classified into three groups: pre-tRNA fragments from the 5´ or 3´ portions of pre-tRNAs, tRNA halves that are cleaved in the anticodon loop of mature tRNAs, and tRNA-derived small RNAs (tsRNA) around 18-23nt from 5´ or 3´ of mature tRNAs [9,10].

As key components of the translation system, tRNAs are highly regulated during the steps of maturation, modification, and trafficking [11]. Under normal growth condition of mammalian cells, high levels of aminoacylation of mature tRNAs are maintained through interactions with their cognate aminoacyl-tRNA synthetases (ARSs), which charge a specific tRNA with the correct amino acid, and translation elongation factors [12]. While in mammalian cells, most of the mature tRNAs are complexed with ARSs, elongation or initiation factors and ribosomes, certain tRNAs are fragmented and released from the protein synthesis cycle [13]. The accumulation of tRNA halves by angiogenin under stress conditions, interaction with Y-box-binding protein 1 (YBX1), and the inhibition of translation process by tRNA halves have been observed for years and studied extensively [3,14–16]. In contrast, the biogenesis and regulation of tsRNAs, especially 3´tsRNAs, remain largely elusive. The exploration of the tsRNA biogenesis pathways and the identification of the tsRNA interaction partners require further characterization of the tsRNAs.

Previously, we have shown that the 22nt 3´tsRNA, LeuCAG3´tsRNA, is required for the efficient translation of several ribosomal protein mRNAs [17]. Particularly, tsRNA^Leu^ is shown to facilitate the ribosomal protein RPS28 mRNA translation by pairing and altering the secondary structure of the mRNA. Northern blot results, sequencing and bioinformatic analysis showed that LeuCAG3´tsRNA is a 22nt small RNA containing the universal CCA terminal sequence at its 3´ end. Unlike the tRNA halves, LeuCAG3´tsRNA can be detected in mammalian cells under normal growth conditions, when most of the tRNA^Leu^ is charged [12]. Thus, the charging status of tsRNA^Leu^ may offer a hint to their biogenesis and regulation. Nevertheless, several traditional methods used for the analysis of tRNAs are not feasible when applied to characterize the charging status of 3´tsRNAs [18–20]. One reason is that the acid urea polyacrylamide gels that can separate charged tRNA from uncharged tRNA in size are not successfully used for detecting tsRNAs, as a result of their low abundance *in vivo* and the relatively lower sensitivity in this type of gel in a northern assay. Secondly, the periodate oxidation, which causes a 1-2nt shortening of the 3´ uncharged tRNA in urea acrylamide gels, leads to the inevitable degradation of a small amount of tRNA. While these methods are not problematic in a tRNA study, it is challenging to differentiate the degraded tRNA portion from the endogenous tsRNA.

Here, we report a new method employing the enzyme exonuclease T, which specifically cleaves the–A residue off the unprotected–CCA at the 3´ end of tsRNAs, leaving the charged–CCA end intact by virtue of the acetylated protection of the 3´-aminoacyl group [21]. To begin to explore the implication of the tRNA charging status on tsRNA biogenesis, we studied the roles of the ARSs in regulating the formation of tsRNAs. We used various approaches to lower cytoplasmic leucyl-tRNA synthetase (LARS1) levels and/or activity in HeLa cells to alter the charging ratio of tRNA^Leu^ and evaluated their effects on tsRNA^Leu^ charging.

## Results

### Exonuclease T cleaves 3´ end adenosine from uncharged tRNA, whereas *N*-acetylated aminoacyl-tRNAs are protected

**Fig 1:**
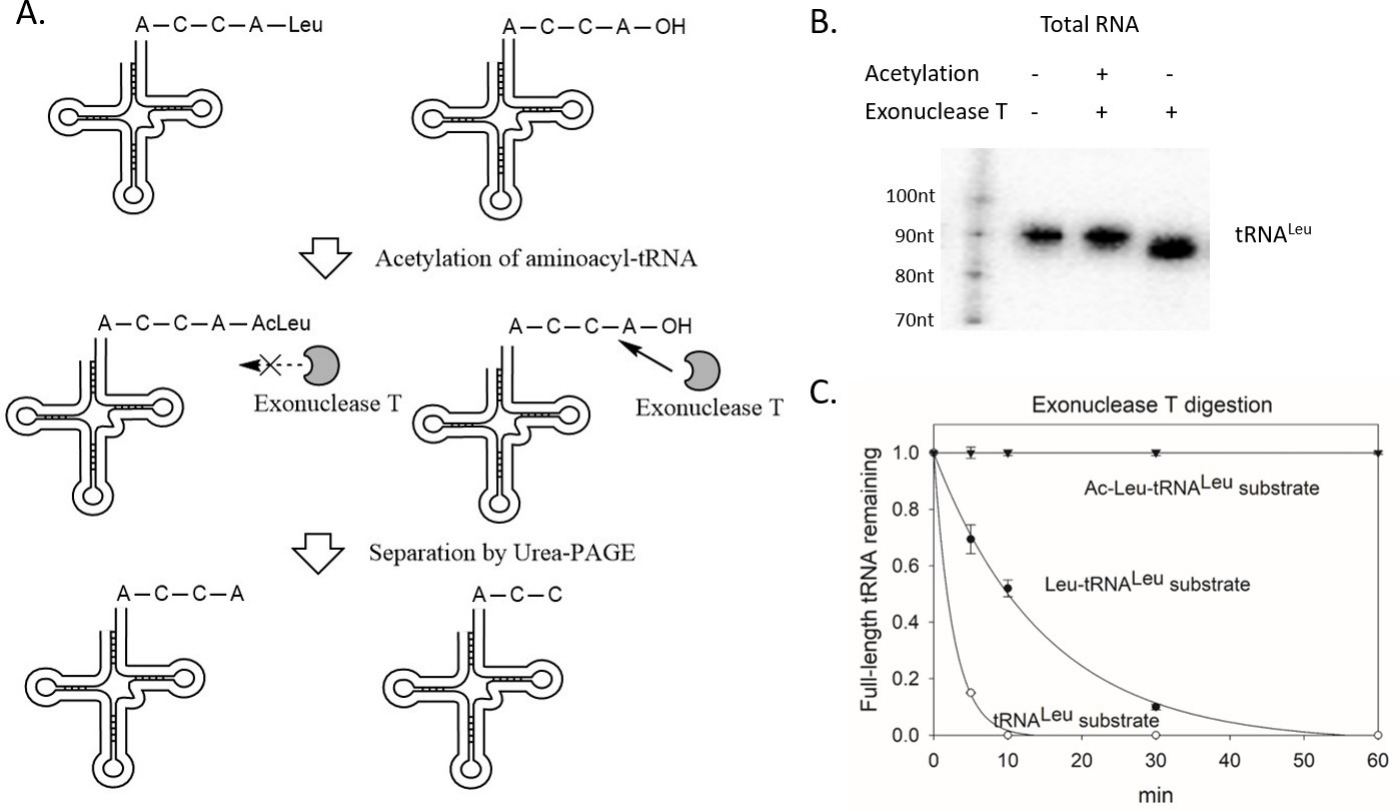
Exonuclease T digests the nucleotide A from 3´-end of uncharged tRNA. (A) Illustration of the enzymatic method to differentiate charged and uncharged tRNAs. (B) Uncharged tRNA^Leu^ digested by exonuclease T is 1 nt shorter than charged tRNA^Leu^ in a northern blot. Total RNA from HeLa cells was treated with or without *N*-acetylation, with or without exonuclease T digestion, and loaded on a 15% urea acrylamide gel. Radiolabeled RNA marker with various sizes were loaded in the left lane. A northern blot was performed using the tRNA^Leu^ probe. (C) *N*-acetylation protects Leu-tRNA^Leu^ from deacylation and digestion with exonuclease T treatment. *E*. *coli* acetylated-Leu-tRNA^Leu^ (inverted triangle). Leu-tRNA^Leu^ (solid circle), uncharged tRNA^Leu^ (open circle) were prepared and subjected to exonuclease T digestion at room temperature. Samples were taken and quenched on ice at time points of 0, 5, 10, 30, 60 mins. Northern blots were used to detect the 1nt size difference after digestion. Levels of digested versus undigested tRNAs were quantified by Quantity One with three independent replicates.

To test the charging status of a 3´tsRNA, exonuclease T was used under nearly neutral pH conditions to differentiate charged tsRNAs from uncharged tRNAs on a polyacrylamide urea gel. As shown in Fig 1A, total RNAs extracted under acidic pH from HeLa cells were separated into two aliquots. The RNA in one aliquot was *N*-acetylated. The *N*-acetylation of the amino acid group attached to the tRNA by acetic anhydride stabilizes the aminoacyl bond and prevents the spontaneous deacylation reaction [22] allowing protection of 3´ end at a higher pHs. The other aliquot was incubated under pH 9.0 as commonly used in the tRNA deacylation reaction. After the *N*-acetylation or the deacylation of the aminoacyl group, total RNA was treated with exonuclease T, which specifically digests single-stranded RNA from an unprotected 3´ end and stops at the -CC sites. Thus, uncharged tRNAs were one nucleotide shorter than the charged tRNAs after enzymatic digestion. Indeed, the tRNA^Leu^ band in the deacylated RNA sample ran 1nt lower on an acrylamide gel than the tRNA^Leu^ band in the *N*-acetylation protected RNA sample as shown in Fig 1B. Without the *N*-acetylation protection, the aminoacylated tRNAs were gradually deacylated during the 3´ digestion with exonuclease T. As shown in Fig 1C, *E*. *coli* leucyl-tRNA prepared by *E*. *coli* ARSs was deacylated within 30 mins and digested by exonuclease T thereafter, whereas the acetylated *E*. *coli* leucyl-tRNA remains intact after 60 mins. Taken together, this enzymatic method combining characteristics of exonuclease T and *N*-acetylation protection could be used to examine the 3´ status of a tRNA or tRNA fragment containing a 3´-CCA end.

### The tsRNA^Leu^ is fully charged regardless of the tRNA^Leu^ charging status

**Fig 2:**
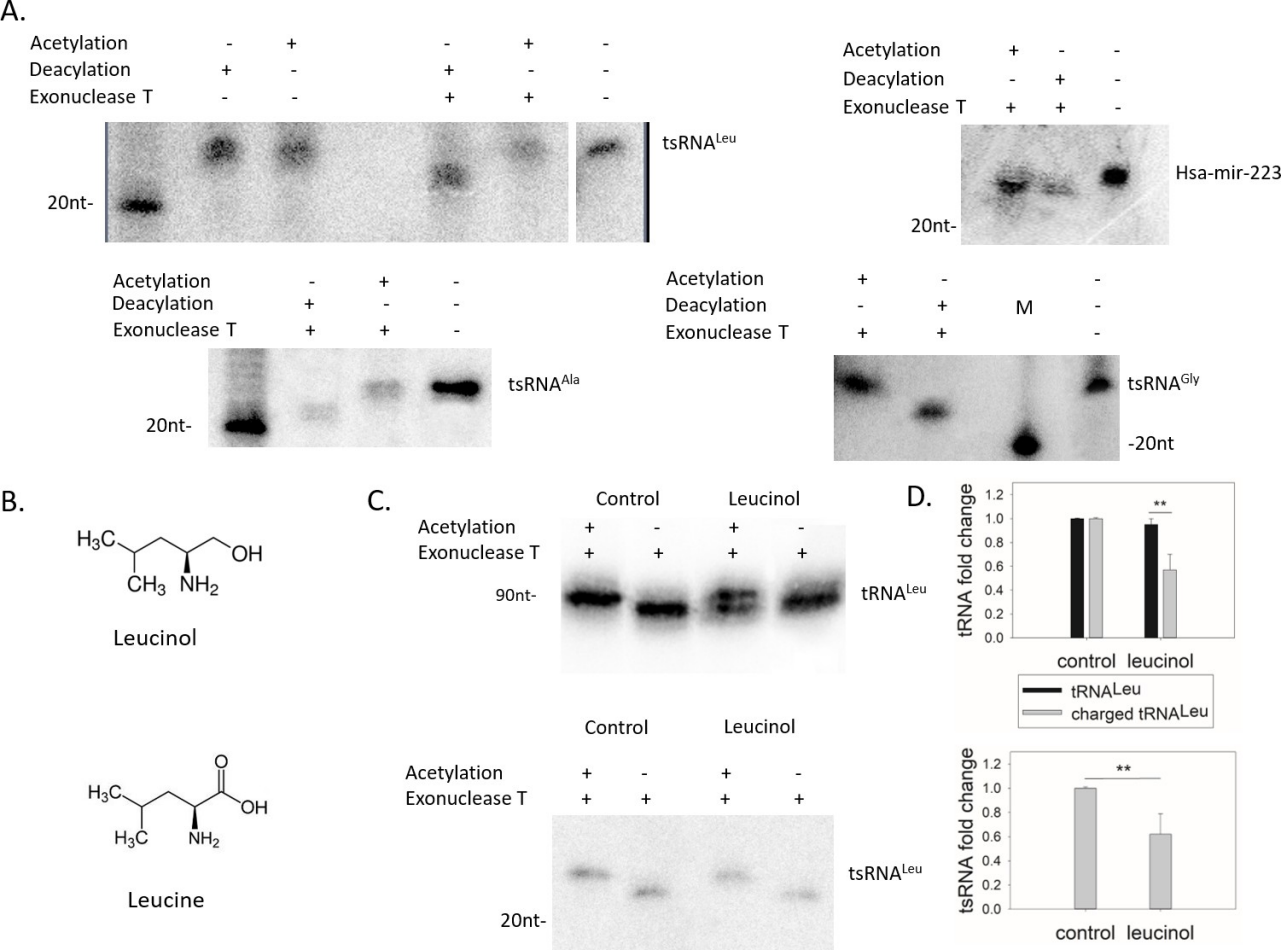
3´tsRNAs are fully charged. All experiments were performed in triplicate and representative northern blots are shown. (A) Under normal growth conditions, 3´tsRNAs are aminoacylated. HeLa total RNA extracted under conditions with pH less than 5.0 was either acetylated or deacylated, ethanol precipitated, and exonuclease T digested. A 20 nt size marker was loaded in each gel and total RNA without any treatment was used for comparison in the right lane. DNA probes antisense to tsRNA^Leu^, tsRNA^Ala^, and tsRNA^Gly^ were used in a northern blot (top left panel and bottom panel). The hsa-miR-223-3p miRCURY LNA miRNA (Qiagen) detection probe was used for quantifying this miRNA (top right panel). (B) Leucinol is an analog of leucine and an inhibitor of LARS1. (C) The tsRNA^Leu^ is fully charged in the presence of leucinol. Total RNA isolated from HeLa cells grown with or without leucinol (2 mM) were extracted and examined for their charging status. The full-length tRNA^Leu^ was ~50% charged under this condition (top) while the tsRNA^Leu^ is fully charged (bottom). (D) Quantification of tRNA^Leu^ and tsRNA^Leu^ in the presence or absence of leucinol. Values were normalized to control cells. Data are means ± standard deviations (s.d.), n = 3 independent experiments, **, P < 0.01, as determined by Student’s one-tailed t-test.

Utilizing our enzymatic method, we successfully characterized the charging status of several tsRNAs. The positive control for the exonuclease T activity in the isolated RNA was the hsa-mir-223 miRNA containing a 3´-CCA that was as expected shortened by one nucleotide in exonuclease T but not control-treated samples. As shown in Fig 2A, all three 3´tsRNAs—tsRNA^Leu^, tsRNA^Ala^ and tsRNA^Gly^ tested were fully charged under normal cell growth conditions.

To investigate whether the tRNA^Leu^ charging level affects the tsRNA^Leu^ charging level, HeLa cells were treated with leucinol, a leucyl-tRNA synthetase aminoacylation inhibitor. Leucinol is a leucine mimic (Fig 2B) that binds to the active site of LARS1 and competes with leucine for aminoacylation [23,24]. Adding leucinol to HeLa cell culture resulted in a ~50% reduction in tRNA^Leu^ charging while the tsRNA^Leu^ was reduced by the same amount but remained fully charged under these conditions (Fig 2C and 2D). Accordingly, the fact that tsRNAs are fully charged even when the tRNA charging is reduced indicates that the tsRNA^Leu^ is generated from the mature tRNA after the mature tRNA is charged by the aminoacyl-tRNA synthetase.

### The tsRNA^Leu^ level is regulated by LARS1

**Fig 3:**
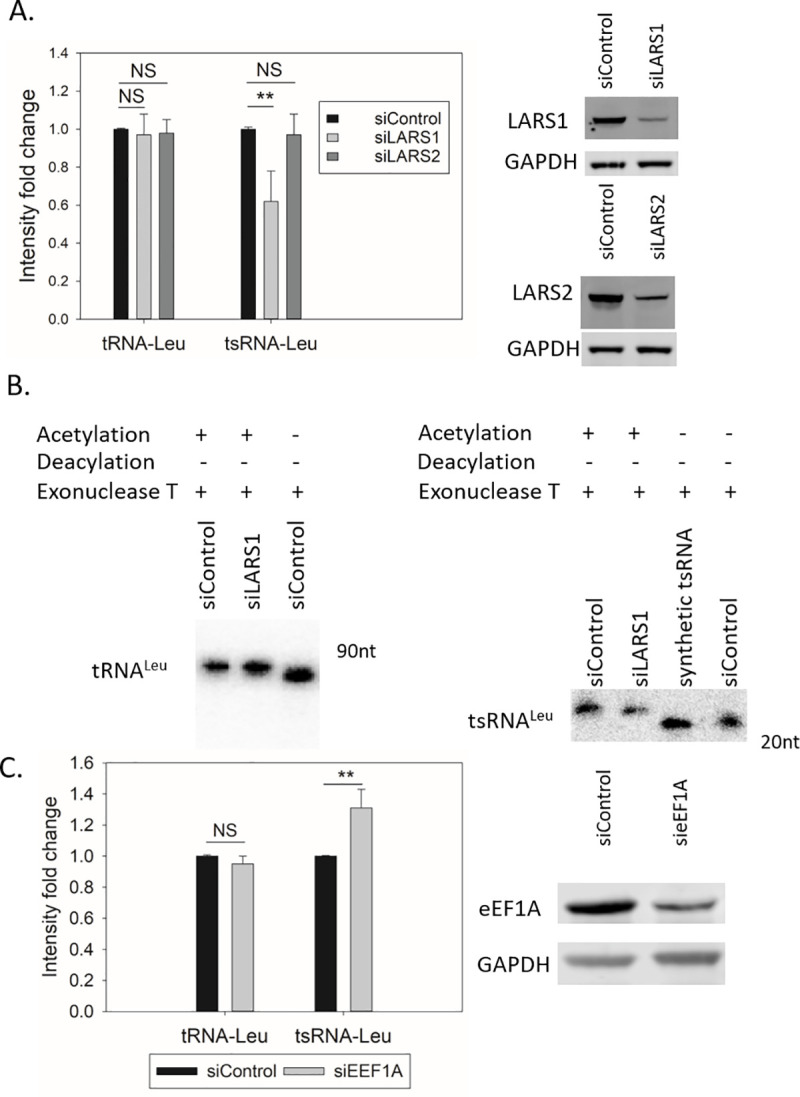
Knockdown of LARS1 decreases tsRNA^Leu^. (A) siRNAs targeting either LARS1 or LARS2 were transfected into HeLa cells in a 6-well plate. Total RNA was extracted after 72 hours, and northern blots were performed to quantify the tRNA^Leu^ and tsRNA^Leu^ levels. Values are presented as means ± s.d. (n = 9). **, P < 0.01, NS, not significant, as determined by Student’s one-tailed t-test. Western blots were used to estimate the concentration of LARS1 and LARS2 protein. (B) tRNA^Leu^ and tsRNA^Leu^ are both fully charged in cells transfected either with LARS1 siRNA (siLARS1) or negative control siRNA (siControl). A chemically synthetic 22-mer tsRNA oligo was treated and used as a size control. (C) siRNA control or targeting eEF1A were transfected to HeLa cells and total RNA was extracted 72 hours later. Northern blots were performed to quantify the tRNA^Leu^ and tsRNA^Leu^ levels. Values are presented as means ± s.d. (n = 3). **, P < 0.01, NS, not significant. Western blots were performed to quantify eEF1A protein levels.

To explore the effect of leucyl-tRNA synthetase (LARS) on tsRNA formation, we used siRNAs to separately reduce the expression of cytoplasmic LARS1 or the mitochondrial LARS2, and then determined the tsRNA levels in each case. As shown in Fig 3A, knockdown of LARS1, but not LARS2, led to a reduction in the tsRNA^Leu^ level while the tRNA^Leu^ levels remained the same. Furthermore, even with siRNA mediated knockdown of LARS1 both the tRNA^Leu^ and 3´tsRNA present in the cells remained fully charged (Fig 3B). The sole reduction in the concentration of the 3´tsRNA (albeit fully charged) with less LARS1 activity strongly implies that the generation of the tsRNAs are regulated by the corresponding ARS complex.

Since charged tRNA^Leu^ is delivered from the LARS1 to eEF1A protein, and eEF1A facilitates the release of charged tRNA from the aminoacyl synthetase, we knocked down eEF1A and quantified the tsRNA concentration. By reducing eEF1A, there was a 30% increase in the tsRNA level (Fig 3C). These results together with the decrease in tsRNA level with less LARS1 protein suggest that the concentration of tsRNA^Leu^ can also be altered by mechanisms unrelated to the absolute concentration of tRNA^Leu^, and further supports the idea that LARS1 is part of a regulated tsRNA^Leu^ biogenesis pathway. The fact that the tsRNA^Leu^ level is regulated through LARS1, but not LARS2, correlates with the exclusive cytoplasmic localization of 3´tsRNAs [10].

### Overexpressing tRNA^Leu^ charging variants give rise to fully charged tsRNA^Leu^

**Fig 4:**
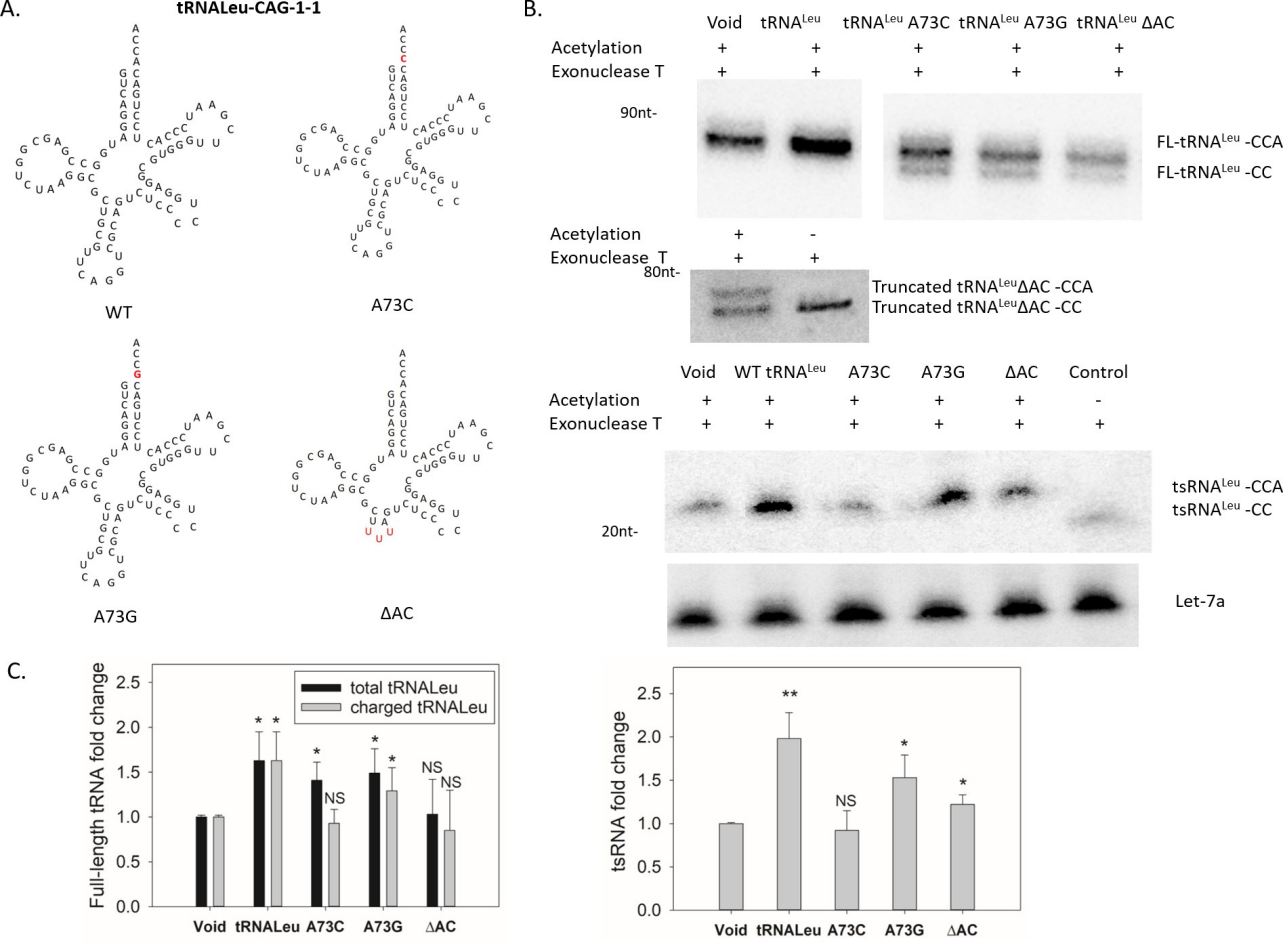
Overexpressions of chargeable or unchargeable tRNA^Leu^ molecules give rise to fully charged tsRNA^Leu^. (A) Sequence and secondary structure of human tRNA^Leu^ and variants. (B) Charging levels of tRNA^Leu^ and tsRNA^Leu^ in the HeLa cells transfected with plasmids carrying various tRNA^Leu^ mutations. ΔAC stands for the tRNA^Leu^ truncated variant lacking the anticodon domain. Overexpression of ΔAC variant leads to extra truncated tRNA bands around 70nt size in the northern blot as shown in the middle panel. Let-7a-3p was used as a loading control. (C) Quantification of full-length tRNA^Leu^, charged full-length tRNA^Leu^ and tsRNA^Leu^ levels when various tRNA mutants were overexpressed. Values are presented as means ± s.d. (n = 4). *, P < 0.05, **, P<0.01, NS, not significant, as determined by Student´s one-tailed t-test.

We wanted to determine if impaired tRNA charging could affect the generation of tsRNAs. To do this, we constructed various tRNA^Leu^ plasmids that express high levels of tRNA^Leu^ variants with mutations in the identity elements known to affect tRNA^Leu^ charging (Fig 4A). To measure the charging status of these tRNAs, we first tested the overexpression of the wild-type tRNA^Leu^. We performed northern blots with and without exonuclease T treatment to quantify the tRNA^Leu^ and the tsRNA^Leu^ concentrations, and determine their charging levels. Significantly enhanced expression of both the tRNA^Leu^ and the tsRNA^Leu^ with wild-type tRNA^Leu^ overexpression was observed compared with the void control (empty vector) (Fig 4B and 4C). Consistent with results under normal growth conditions, both the tRNA^Leu^ and the tsRNA^Leu^ were fully charged when the wild-type tRNA^Leu^ was overexpressed.

Subsequently, each plasmid carrying the tRNA variant gene was transfected separately and data were obtained similarly as in the wild-type overexpression experiments. Human tRNA^Leu^ A73, as reported previously, was known to significantly affect the LARS1 recognition. Among the human tRNA^Leu^ A73 mutations, the A73C mutant cannot be charged by LARS1 *in vitro*, and the uncharged tRNA^Leu^ A73C mutant also inhibits the aminoacylation of wild-type tRNA^Leu^ [25]. As shown in Fig 4B, overexpression of tRNA^Leu^ A73C resulted in an increased amount of uncharged tRNA^Leu^ yet there was no change in the tsRNA charging level.

Another mutant tRNA^Leu^ A73G was previously shown to confer serine acceptance by seryl-tRNA synthetase (SerRS) instead of LARS1 *in vitro* [26]. Transfection of tRNA^Leu^ A73G resulted in a slight albeit statistically significant increase in the total and charged tRNA^Leu^ versus the control cells (Fig 4C). Similar to the A73C transfection, a small amount of uncharged tRNA^Leu^ was observed (Fig 4B), possibly due to the impaired aminoacylation efficiency by SerRS compared to the cognate LARS1. Again, the tsRNA^Leu^ remained fully charged, suggesting the uncharged tRNA^Leu^ did not give rise to the tsRNA^Leu^ molecule. Additionally, an increase in the tsRNA concentration was observed with A73G overexpression compared to the A73C mutant (Fig 4C), indicating the Ser-tsRNA^Leu^ could also be generated from the mischarged tRNA^Leu^. At this stage, we cannot determine the ratio between the Ser-tsRNA^Leu^ and the Leu-tsRNA^Leu^, as the northern blots cannot differentiate these two aminoacyl groups. It is possible that the mischarged tRNA^Leu^ has a greater likelihood of being cleaved by the nuclease responsible for tsRNA generation because of the altered interaction between the aminoacyl-tRNAs and the responsible ARSs. Alternatively, the elevation of tsRNA level may have resulted from the inherent differences between LeuRS and SerRS during tsRNA biogenesis.

To clearly differentiate the overexpressed tRNA from the endogenous tRNA, a plasmid encoding a truncation mutant of tRNA^Leu^ lacking the anticodon loop was used for transfection. In this case, the ΔAC tRNA^Leu^ was not efficiently charged *in vivo* (Fig 4B, middle panel), despite the previous implications that the tRNA^Leu^ anticodon loop is dispensable for the human LARS1 aminoacylation [26]. This impaired aminoacylation phenomenon is similar to the anticodon loop deletion mutant in *E*. *coli* [27], possibly due to the differences in the hLARS1 charging activity between living cells and a cell-free system. Whereas there was no change in the full-length tRNA^Leu^ level with ΔAC tRNA^Leu^ overexpression, a slight increase of the tsRNA concentration was observed as shown in Fig 4C, suggesting the tsRNA^Leu^ could be generated from the truncated ΔAC tRNA^Leu^. Moreover, the tsRNA^Leu^ was still fully charged with ΔAC tRNA^Leu^ overexpression (Fig 4B). These observations are consistent with the hypothesis that the tsRNA^Leu^ originated from charged tRNAs. Taken together, modulations of the tRNA and tRNA variants levels can regulate the tsRNA concentration and give rise to the fully aminoacylated tsRNAs.

### Bioinformatic analysis of tsRNA^Leu^ in small RNA sequencing libraries

**Fig 5:**
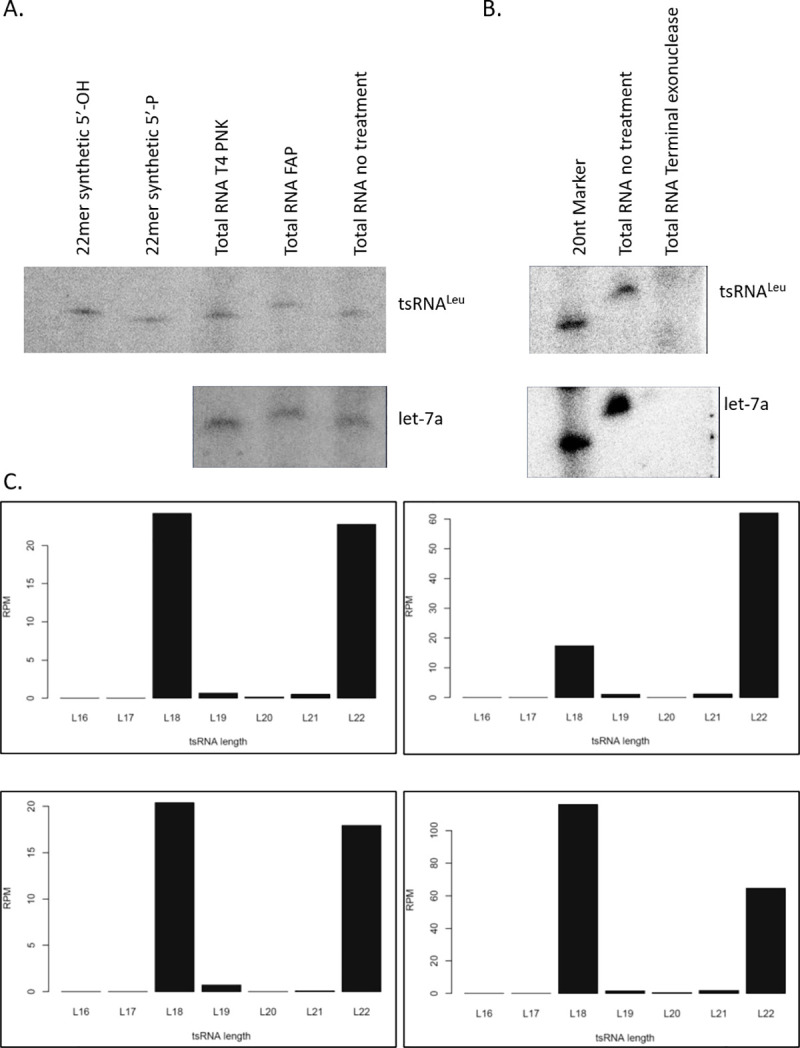
5´ terminal 3´ tsRNA^Leu^ is phosphorylated. (A) HeLa total RNA was treated with T4 PNK or FAP and northern blots were performed. Nontreated RNA sample, synthetic 22mer tsRNA^Leu^ with either a 5´-hydroxyl or 5´-phosphate group were used as controls to indicate the upshift of tsRNA^Leu^ after FAP treatment. The miRNA let-7a serves as a control RNA with 5´-phosphate. (B) Terminal exonuclease digested the tsRNA^Leu^ as well as let-7a with the 5´-phosphate. (C) Length distribution of reads that mapped to 3´ end of tRNA-Leu-CAG-1-1. TsRNA^Leu^ reads from HeLa small RNA libraries GSM876014, GSM876013, GSM876017, GSM876016.

To evaluate the tsRNA level from high-throughput sequencing databases, the phosphorylated status of the 5´ end of tsRNA^Leu^ was examined by several enzymatic methods. As shown in Fig 5A., total RNA was treated with either T4 polynucleotide kinase (T4 PNK) or fast alkaline phosphatase (FAP), and the mobility of the tsRNA^Leu^ bands was determined by northern blot. The FAP treatment was able to remove the 5´ phosphate group of tsRNA and shift the band, whereas the T4 PNK treatment that phosphorylates the 5´ end of RNA did not affect the band position. In addition, the terminal exonuclease that is able to digest only 5´ phosphorylated RNAs removed the tsRNA^Leu^ band as shown in Fig 5B. Similar results were observed using a let-7a miRNA probe. Taken together, these results suggest that the 5´ end of tsRNA^Leu^ is phosphorylated and would be amenable to 5´ adaptor ligation used in high-throughput sequencing.

In most RNA sequencing libraries, tRNA reads may not be accurately quantified because the reverse transcriptase tends to stop at various modification sites and/or by structural elements, resulting in truncated tRNA reads. To exclude the truncated reads from full-length tRNAs, only small RNA sequencing libraries with size selection between 17–30 bases were explored for our study. We analyzed four different small RNA libraries from HeLa cells and aligned the reads to the tRNA-Leu-CAG-1-1 gene according to the hg19 genomic tRNA database [28]. As shown in Fig 5C, results from all sequencing databases showed two specific peaks with a similar number of reads in each sample, representing the 22nt full-length tsRNA^Leu^ and the 18nt truncated tsRNA, the latter, which was not detected by northern blot [17]. Although most truncated reads in deep sequencing are thought to be from reverse transcriptase stops at the modification site, we cannot rule out the possibility that there are different levels of degradation of tsRNA at the 18nt modification (m1A) site during the adaptor ligation steps. In either case, the sum of the two peaks can be calculated to determine the relative tsRNA^Leu^ concentration. In addition, the construction of small RNA libraries in the past seldom involves the tRNA deacylation step. It is possible that the 3´ end of the tRNAs and tsRNAs would not be ligated to the adaptor due to the presence of the aminoacyl bonds at the time of library preparation. In summary, determining the correlation of the 22-nt tsRNA with cellular status by high throughput sequencing method requires further evaluating tRNA modification levels and developing optimized tRNA-seq protocols including removing modifications and eliminating the ligation [29–33].

## Discussion

**Fig 6:**
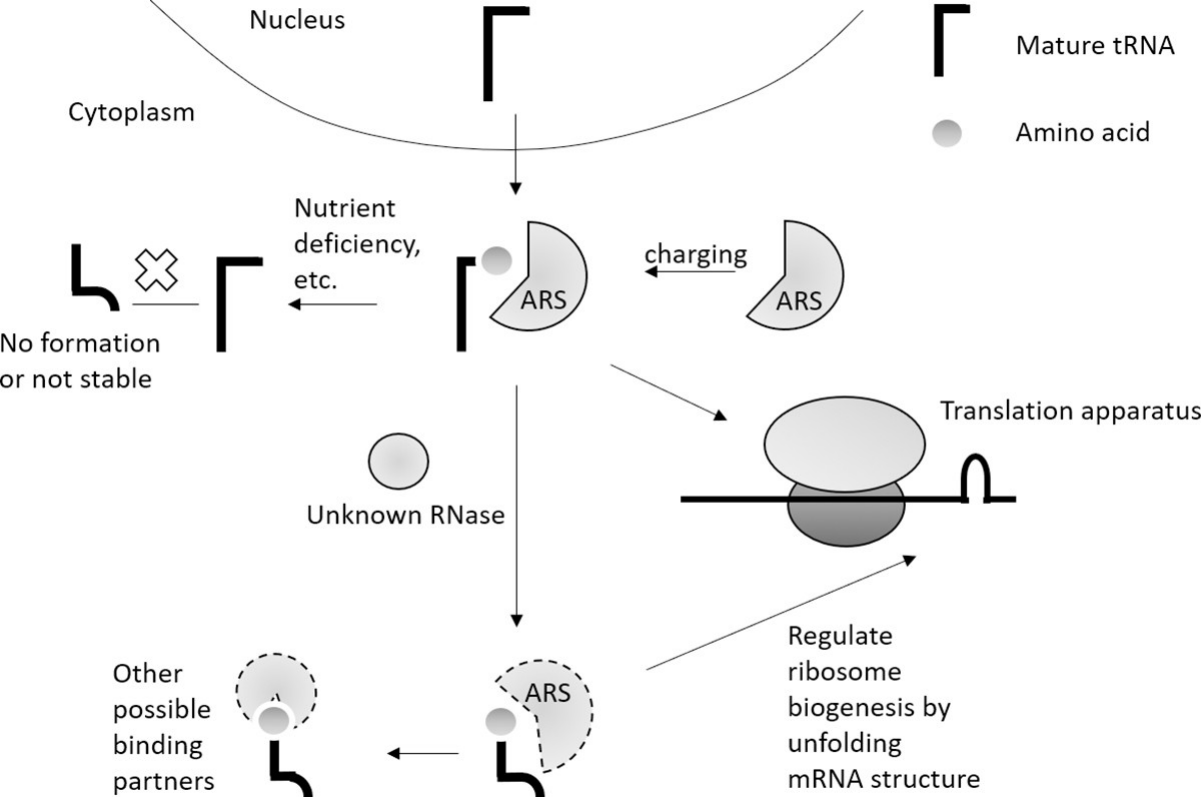
3´tsRNA were generated from cytoplasmic aminoacyl-tRNAs and regulated by aminoacyl-tRNA synthetases (ARSs).

As a novel class of small ncRNAs discovered in recent years, the reporting functions of the tsRNAs in translational control, oncogenesis, and neurologic disorders are emerging [13]. Dysregulation of tsRNAs have been found in various cancers and diseases [1,34], and in order to gain some insight into how, where and when they are generated, we sought to determine if the 3´tsRNAs are aminoacylated. Because of the low abundance of 3´tsRNA and instability of the phosphodiester bond in neutral or high pH solutions and elevated temperature in beta elimination treatment, characterizing the charging status of 3´tsRNA^Leu^ has been difficult. Here we developed a sensitive enzymatic method to examine the charging status of 3´tsRNA^Leu^ under various cellular conditions. Elucidating the fact that 3´tsRNA^Leu^ is fully charged regardless of the mature tRNA charging level not only suggests that tsRNA^Leu^ is generated from mature aminoacyl-tRNA, but also indicates the interaction of tsRNAs with ARSs (Fig 6). The fact that tsRNAs are charged and their concentration regulated by tRNA synthetases levels, as well as other interacting partners suggests that the 3´tsRNA biogenesis is a highly regulated process. In total, our study characterizing the terminal status of tsRNA, together with manipulation of aminoacyl-tRNA synthetases, provide new insights into the generation of 3´tsRNAs.

Since it has been shown that tsRNAs from the 3´ end of tRNAs are localized in the cytoplasm [10], it is not surprising that tsRNA^Leu^ is charged under normal growth conditions when most tRNAs are highly aminoacylated [12]. Nonetheless, lowering the aminoacylation level of tRNA^Leu^ by 50% with leucinol still gives rise to fully charged tsRNAs. This result indicated that tsRNA^Leu^ originates from mature tRNAs and the processing of tsRNA^Leu^ happens after aminoacylation by ARSs. Further evidence came from *in vivo* overexpression of wildtype tRNA^Leu^ and various mutants. While overexpression of the wild-type tRNA-Leu-CAG-1-1 gene elevated the level of charged tsRNA^Leu^ compared to the control, overexpression of the unchargeable mutant, A73C resulted in no change or a slightly lower amount of aminoacyl-tsRNA. The effect of A73C overexpression results from the inhibition of wild-type tRNA^Leu^ aminoacylation induced by the presence of A73C tRNA^Leu^. In comparison, overexpression of the chargeable mutant A73G tRNA^Leu^ led to an increase in the tsRNA levels albeit to a lower extent compared with the wild-type tRNA gene. A73G tRNA^Leu^ was shown to be mischarged by SerRS *in vitro*, suggesting the biogenesis of this tsRNA may not be limited by the fidelity of tRNA aminoacylation. In addition, by deleting the anticodon loop of tRNA^Leu^, we established that the ΔAC tRNA^Leu^ is charged to 50% by LARS1 *in vivo* and gave rise to increased tsRNA concentrations. Taken together, these data strongly support that the 3´tsRNA^Leu^ is generated only from aminoacylated tRNAs, and that the concentration of chargeable tRNAs might at least in part regulate the production of 3´tsRNA.

tRNA charging occurs via ARS proteins in a poorly defined complex referred to as the multi-aminoacyl-tRNA synthetase complex (MSC) consisting of multiple proteins including elongation factor 1A. This along with a number of studies have shown that the MSC is associated with actively translating ribosomes [35]. Reducing LARS1 did not change the level of aminoacyl-tRNA^Leu^ but rather resulted in a decrease in the tsRNA^Leu^ level, suggesting that the tsRNA^Leu^ level might be regulated by aminoacyl-tRNA synthetases. It is therefore possible that the corresponding nuclease for tsRNA biogenesis binds with ARSs to regulate their production. The fact that the tsRNA concentration was increased while the tRNA level was unchanged after eEF1A knockdown is consistent with this hypothesis. Another possibility is that the ARSs act as chaperones and protect the charged tsRNAs from degradation. Evidence of tsRNA binding with ARSs supported the possibility that at least a portion of the tsRNAs is contained near the translation apparatus [36]. The finding that tRNA fragments are associated with ARSs, but not EF1A also supports the idea that tRNA-derived small RNAs are regulated by ARS complexes [37].

For the reason that the 3´tsRNA^Leu^ regulates ribosome biogenesis by regulating the production of a key ribosomal protein, RPS28 [17,38], the ARS complexes on actively translating ribosomes may indeed function in some sort of feedback regulatory mechanism. It is possible that the ribonucleases responsible for tsRNA formation interact with the aminoacyl-tRNA synthetases and/or ribosomes to regulate the ribosome biogenesis and other cellular processes as part of this feedback mechanism. Increased concentrations of 3´tsRNA^Leu^ were found in both murine and human hepatocellular carcinomas, which when blocked induced apoptosis and shrinkage of the tumors and hence a possible target for cancer treatment. Furthermore, adding 3´tsRNA^Leu^ mimics to cells increased RPS28 protein [17] suggesting that this tsRNA may be important perhaps in increasing the number of ribosomes that are required for enhanced protein synthesis in conditions such as cancer. The fact that ribosome biogenesis can consume over 60% of the cellular energy [39] makes it likely there are as of yet unknown mechanisms for fine-tuning the production and assembly of the protein-synthesizing machinery.

It has been reported that tRNA fragments can be generated by various RNases, such as RNase T2, angiogenin (ANG), and Dicer [40,41]. Nevertheless, these enzymes are responsible for the formation of only certain species of tRNA fragments. Specifically, the nuclease responsible for the biogenesis of the 22mer 3´tsRNAs from mature tRNAs has yet to be determined. Firstly, human RNase T2 was shown to digest ssRNA between purine and uridine residues, leaving the RNA fragments ending with 2´, 3´-cyclophosphate configurations [42]. The fact that the 3´tsRNAs are cleaved between T54 and Ψ55 and their 5´-ends are phosphorylated suggests a mechanism other than RNase T2. Secondly, a previous enzymatic study had shown that ANG could generate tRNA fragments but differed in size from the 22-mer tsRNAs [43]. Recently a knock-out study also supported that angiogenin was responsible for certain species of tRNA halves, but not for the 3´tsRNAs [44]. Thirdly, as a double-strand endonuclease, Dicer was shown to cut tRNA mainly at the double-stranded T-arm region and minimally at the T-loop [45]. Indeed, though the levels of several 3´tRNA fragments were shown to be Dicer-dependent, there were no differences in the level of other tsRNA species, such as LeuCAG3´tsRNA fragments, in knock-out cells [43]. Taken together, defining the cleavage mechanism for the generation of 3´tsRNAs requires further characterization of alternate RNases. It is possible that multiple steps and enzyme pathways are involved in the biogenesis of tsRNA.

Changes in tRNA post-transcriptional modification levels are reported to affect the biogenesis of tRNA fragments. It has been reported that lack of cytosine-5 methylation from DNMT2 or decreasing m1A and m3C levels by ALKBH3 could promote the production of tRNA halves by angiogenin [4,46]. In addition, the deficiency of TRMT10A, a guanosine 9 tRNA methyltransferase, led to the fragmentation of tRNA^Gln^ and tRNA^iMet^ in pancreatic β-like cells [47]. Recently, an RNA phospho-methyltransferase BCDIN3D was reported to interact with the tRNA^His^ 3´ fragments and regulate their biogenesis [45]. It is possible that the RNA modifying enzymes regulate the tsRNA generation by affecting the stability of the tRNA structure and the interaction among the tRNA, aminoacyl-tRNA synthetase complex and ribosomes. The nuclease responsible for 3´tsRNA formation likely binds to the aminoacyl-tRNA synthetases in a specific structural orientation and thus cleaves only the elbow region of the charged tRNAs. Therefore, the enzymes responsible for Ψ55 pseudouridine and other tRNA modifications on this region could also be candidates for tsRNA regulation. Further identification of tRNA and tsRNA modifying proteins may provide more evidence for the highly variable, cell-specific biogenesis of tsRNAs.

## Materials and methods

### Cell culture and transfection

HeLa cells were cultured in Dulbecco´s modified Eagle´s medium supplemented with 10% fetal bovine serum in a 5% CO_2_ incubator. For RNA interference, Opti-MEM medium and Lipofectamine 3000 (ThermoFisher) was used as per the manufacture´s protocol. HeLa cells were transfected with control siRNA (non-targeting pool, Dharmacon) or siRNAs targeting human leucyl-tRNA synthetase 1 or 2 (Dharmacon), separately. For transfection of tRNA plasmids, HeLa cells were transfected by Lipofectamine 3000 with 2 μg of plasmid encoding tRNA wild-type or variant genes and incubated for 24 hours before RNA extraction.

### Construction of tRNA expression plasmids

The gene encoding human cytosolic tRNA-Leu-CAG-1-1 was cloned with 150 bp 5´ and 3´ flanking regions from the genome. The corresponding tRNA^Leu^ region was inserted into pUC19 plasmid (ptRNA^Leu^CAG). Mutation of A73 to G or C, and the anticodon stem deletion in the tDNA genes was made by QuickChange lightning site-directed mutagenesis kit (Agilent) using the following primers:

**Table:** 

CAG-AtoG_F	ATCCCACTCCTGACGATATGTGTTTTCCGT
CAG-AtoG_R	ACGGAAAACACATATCGTCAGGAGTGGGAT
CAG-AtoC_F	ATCCCACTCCTGACCATATGTGTTTTCCGT
CAG-AtoC_R	ACGGAAAACACATATGGTCAGGAGTGGGAT
CAG-delta_AC_F	GGTCGCAGTCTTTTGGCGTGGGTTC
CAG-delta_AC_R	GAACCCACGCCAAAAGACTGCGACC

### RNA isolation, N-acetyl protection and deacylation of tRNA, and exonuclease T treatment

Cells were harvested using trypsin and washed with cold phosphate-buffered saline. Total RNA was extracted by Trizol (Invitrogen) and Direct-zol RNA kit (Zymo Research). RNA was kept in buffers at pH 5 at all times. For the aminoacylation characterizing assay, total RNA was separated into two fractions. One fraction was incubated on ice in 250 μl of 0.2 M sodium acetate with 4 μl acidic anhydride for 2 hours for *N*-acetylation as described previously [48]. The other fraction was incubated at 37 degrees at pH 9.0 for 30 min to deacylated the aminoacyl group [48]. Both fractions were then ethanol precipitated at -80 degrees for 2 hours and washed with 80% ethanol. 10 ug total RNA was resuspended in 87 μl RT-PCR grade water (ThermoFisher), and 3 μl exonuclease T (New England Biolabs, NEB) with 10 μl buffer 4 (NEB) was used in the enzymatic reaction. The digestion reactions were kept at room temperature for 1 hour, and then the RNA clean and concentrator kit (Zymo) was used to remove the exonuclease T and other residues. The RNA samples were quantified by nanodrop (Agilent) and subjected to subsequent applications.

### Northern blot

10 μg total RNA samples were mixed with gel loading buffer II (ThermoFisher) and separated by 10% or 15% TBE-urea gels (Novex) in 1x Tris-borate-EDTA (TBE) buffer at 180 V for 90 min. RNA was transferred to a positively charged nylon membrane (Hybond N+, GE Healthcare) through a semi-dry electrophoretic transfer cell (Biorad) in 0.5x TBE buffer at 5 Watt for 1 hour. UV crosslinker (Stratagene) was used to crosslink RNA to the membranes at 150 mJ/cm^2^. Membranes were prehybridized by PerfectHyb plus (Sigma) hybridization buffer for 1 hour and hybridized overnight with a [γ-32P] ATP-labeled probe that was complementary to 3´ end of tRNA-Leu-CAG-1-1. Membranes were washed with 2x SSC and 0.1% SDS three times for 10 minutes.

### Aminoacyl-tRNA preparation

Aminoacylation was performed in 100 mM Hepes-NaOH (pH 7.6), 30 mM KCl, 10 mM MgCl2, 1 mM dithiothreitol (DTT), 2 mM adenosine triphosphate (ATP), 10 mM Leucine, 1.5 mg/mL of *Escherichia coli* total tRNA (Roche), and 10 nM of crude ARSs from *Escherichia coli* (Sigma, A3646) [48,49]. After a 30 min incubation at 37°C, tRNA was ethanol precipitated at -80 degrees for 2 hours and washed twice with 70% ethanol. The resulted mixture of aa-tRNA and tRNA was separated and analyzed by urea acrylamide gels and northern blots.

### Western blot

Transfected cells were lysed in cell lysis buffer (Cell signaling) with 1x protease inhibitor cocktail (Sigma) on ice for 15 minutes. After centrifugation at 13,000 rpm at 4 degrees for 30 minutes, the precipitant was removed, and protein was quantified using a Pierce BCA assay kit (ThermoFisher). 4 μg protein was resolved on NuPAGE 4–12% Bis-Tris protein gels (ThermoFisher) and transferred to a PVDF membrane (Fisher). The membranes were blocked by Odyssey blocking buffer (Licor) and then immunoblotted with the cytoplasmic LARS1, the mitochondrial LARS2 or GAPDH primary antibodies (Sigma) and IRDye secondary antibodies (Licor). Results were visualized using the Odyssey imaging systems.

### 5´ phosphorylation characterization

Total RNA was extracted by Trizol and treated with FastAP (ThermoFisher), T4 polynucleotide kinase (New England Biolabs, with 1mM ATP) and Terminal exonuclease (buffer A) following their standard protocol. 5´ hydroxyl and 5´ phosphorylated 22mer tsRNA^Leu^ oligos were synthesized by Dharmacon.

### Bioinformatic analysis of 3´-tsRNA

The small RNA deep sequencing datasets for HeLa cell line were downloaded from the GEO database. Only libraries generated from 17–30 bases sized selected small RNAs were analysed. Human tRNA gene sequences (human hg19) were downloaded from the genomic tRNA database (27). Small RNA reads were mapped onto the tRNA gene sequences with CCA end added at the 3´ end using Bowtie version 1.2.2 [50].

## Supporting information

S1 Table Summary of expression levels of tRNAs and tsRNAs under various conditions. (XLSX)
